# Empathy and Coping Strategies Predict Quality of Life in Japanese Healthcare Professionals

**DOI:** 10.3390/bs14050400

**Published:** 2024-05-11

**Authors:** Kotaro Shoji, Norihito Noguchi, Fumiko Waki, Taku Saito, Masato Kitano, Naoki Edo, Minori Koga, Hiroyuki Toda, Nobuhisa Kobayashi, Takehito Sawamura, Masanori Nagamine

**Affiliations:** 1 College of Nursing, University of Human Environments, 3-220 Ebata, Obu 474-0035, Aichi, Japan; k-shoji@uhe.ac.jp; 2 Department of Nursing, National Defense Medical College, 3-2 Namiki, Tokorozawa 395-8513, Saitama, Japan; 3 Division of Behavioral Science, National Defense Medical College Research Institute, 3-2 Namiki, Tokorozawa 395-8513, Saitama, Japan; 4 Department of Psychiatry, National Defense Medical College, 3-2 Namiki, Tokorozawa 395-8513, Saitama, Japan; 5 Department of Psychiatry, Self-Defense Forces Central Hospital, 1-2-24 Ikejiri, Setagaya, Tokyo 154-8532, Japan

**Keywords:** Japanese healthcare worker, coping, burnout, empathy, secondary traumatic stress, compassion satisfaction

## Abstract

Burnout and secondary traumatic stress (STS), also referred to as compassion fatigue, are undeniable negative consequences experienced by healthcare professionals when working with patients. As frontline healthcare professionals are essential to communities, it is crucial to understand their mental health and how they cope with negative psychological responses. This study investigated the relationships between burnout, STS, compassion satisfaction, dispositional empathy, and stress management among Japanese healthcare professionals and students taking care of patients in clinical practice. The participants were 506 Japanese healthcare professionals and students (doctors, nurses, medical students, and nursing students) affiliated with Japanese Ministry of Defense Hospitals. The data were collected from March 2020 to May 2021. We assessed burnout, STS, and compassion satisfaction using the Professional Quality of Life Scale, dispositional empathy using the Interpersonal Reactivity Index, and coping with stress using the Coping Orientation to Problems Experienced Inventory (Brief-COPE). Exploratory factor analysis of the Brief-COPE yielded three factors: active coping; support-seeking; and indirect coping. Personal distress, a self-oriented emotional empathy index, was related to higher burnout and STS scores and lower compassion satisfaction. Empathic concern, an other-oriented emotional empathy index, was associated with lower burnout and higher compassion satisfaction. Active coping strategies were associated with lower burnout and higher compassion satisfaction, whereas indirect coping strategies were associated with higher burnout and STS scores. In a comparison of empathy in professional categories, nurses presented higher personal distress than nursing students, and medical doctors showed lower fantasy tendencies than medical students. These results imply the complex relationships between empathy, coping strategies, and psychological responses among healthcare professionals. Further longitudinal study is needed to explore these complex relationships and to develop more precise and effective psycho-educational interventions to prevent burnout and STS.

## 1. Introduction

Burnout and secondary traumatic stress (STS) are negative consequences experienced by healthcare professionals. Burnout refers to the emotional exhaustion and loss of motivation due to prolonged exposure to chronic stressors [1]. Burnout is prevalent worldwide, especially in clinical settings, with 66.0% of healthcare professionals in Hong Kong experiencing moderate burnout and 4.7% experiencing high levels of burnout [2], 78.5% and 0.0% of Greek healthcare professionals experiencing moderate and high levels of burnout, respectively, during the COVID-19 pandemic [3], and 91% of adult or pediatric healthcare professionals in Japan exhibiting moderate levels of burnout, and 4% showing high levels of burnout [4].

STS, sometimes referred to as compassion fatigue, is characterized by reduced empathic responses to, or lower interest in, clients resulting from indirect trauma exposure through client care [5]. STS involves symptoms like those of posttraumatic stress disorders (i.e., hyperarousal, re-experiencing, avoidance) due to indirect trauma exposure [6]. Healthcare workers who care directly for clients are often exposed to clients’ traumatic experiences; hence, they are at an increased risk of developing STS symptoms. Previous studies have shown that 48.4% and 2.2% of Greek healthcare professionals had moderate or high levels of STS, respectively, during the COVID-19 pandemic, and 89% and 8% of Japanese healthcare professionals working in intensive care units were identified as having moderate and high levels of STS, respectively [3,4].

In contrast, compassion satisfaction refers to the positive feelings healthcare professionals derive from helping others [7]. Individuals with high compassion satisfaction tend to have low burnout and STS scores [8]. However, these associations have small to medium effect sizes, indicating that compassion satisfaction is an independent component of the consequences of helping others.

Healthcare professionals’ psychological responses are associated with empathy. Empathy refers to the capacity to understand other people’s responses to their experiences from their viewpoints and react accordingly [9]. The multidimensional model of empathy posits that empathy comprises emotional and cognitive components [9,10]. The cognitive component of empathy, known as perspective taking, refers to spontaneous attempts to adopt other people’s perspectives and understand others’ feelings, thoughts, and beliefs [9,10]. The emotional component of empathy, known as empathic concern, facilitates the cognitive component, helping observers understand others’ intentions, emotions, beliefs, and motivations [11]. Moreover, personal distress, an additional emotional component, refers to the tendency to feel distress and anxiety in response to exposure to others’ feelings [9]. Fantasy tendency, another sub-component of empathy, refers to the inclination to identify with fictitious characters [9].

Previous studies of healthcare professionals have investigated the relationship between empathy and psychological responses, including compassion satisfaction, burnout, and STS. Personal distress is negatively related to compassion satisfaction and positively related to burnout and STS among healthcare professionals [12,13,14]. Empathic concern among healthcare professionals is positively related to compassion satisfaction and STS [12,13,15]. Given that empathy subscales are associated with both positive and negative psychological responses, human empathy may be a double-edged blade.

The empathy of healthcare professionals is critical because their empathic characteristics are related to their psychological health and the quality of care they provide [16]. Although the importance of empathy in healthcare professionals has been advocated, empathy among medical students and residents is likely to decline over the course of their careers [17,18]. Similarly, empathy tends to decline with age and career progression among nurses and nursing students [19], and their empathy wanes as they interact with an increasing number of patients [20]. Considering that healthcare professionals show positive and negative psychological reactions through empathic interactions with their patients, coping with such stress must also be considered in understanding their declining empathy and psychological health.

Healthcare professionals use numerous coping strategies for negative psychological responses. For example, during the COVID-19 pandemic, healthcare professionals in New York engaged in physical activities and exercise (59%), yoga (25%), meditation (23%), and faith-based activities (23%), mostly to cope with pandemic-related distress [21]. Additionally, a multinational study conducted during the pandemic indicated that up to 60% of vascular surgeons engaged in active coping and self-distraction, and 20% engaged in other avoidant coping strategies [22]. Active coping strategies refer to coping efforts that focus on matters at hand and planning future actions to focus on problem-solving [23]. Active coping strategies are usually effective in reducing burnout among healthcare professionals [24]. Support-seeking coping strategy refers to focusing on seeking emotional support from others to manage stressful situations [25]. Support-seeking coping strategy was not strongly related to anxiety, depression, or burnout among Cypriot healthcare professionals during the COVID-19 pandemic [26]. Furthermore, avoidant coping strategies refer to trying to avoid stressors rather than dealing with them. However, avoidant coping strategies are related to anxiety, depression, and burnout among healthcare professionals [27].

Given the effects of their empathic characteristics and stress coping on the various psychological reactions that healthcare professionals may suffer, a better understanding of these complex relationships can provide crucial insights into maintaining their mental health. To our knowledge, there are no studies investigating these complex relationships across the careers of Japanese healthcare professionals. This study investigated the relationships between psychological responses (STS, burnout, and compassion satisfaction), dispositional empathy, and coping strategies among Japanese medical doctors, nurses, medical students, and nursing students. This study was part of a larger longitudinal study investigating the trajectories of these study variables among early-career healthcare professionals and nursing and medical students. Based on previous findings, we hypothesized that (1) active coping strategies would be associated with lower burnout and STS and higher compassion satisfaction and that (2) dispositional empathy would be related to the above-mentioned psychological responses. Specifically, we expected personal distress to be positively related to burnout and STS and negatively related to compassion satisfaction. In addition, we expected (3) empathy to be higher in nursing students than in nurses, and similar trends would be observed between medical students and physicians.

## 2. Methods

### 2.1. Participants

This study was a baseline survey of a longitudinal study investigating STS, burnout, coping with stress, and dispositional empathy among Japanese healthcare professionals and students working at the National Defense Medical College and Japanese Self-Defense Forces Central Hospitals. Of the 506 healthcare professionals who responded to the assessment, Table 1 presents the participants’ demographic information. Participants were 64.2% women; their mean age was 24.95 years old, and 20.0% of them were married. Moreover, 181 were doctors in their first to sixth year after graduation (35.8%, mean age = 29.06 [SD = 2.14]); 63 were fifth-year medical students actually taking patients in the wards (12.5%, mean age = 23.98 [SD = 0.92]); 123 were first-year post-graduate nurses (24.3%, mean age = 23.47 [SD = 1.29]), and 139 were third-year nursing students actually taking care of patients on the wards (27.5%, mean age = 21.33 [SD = 0.76]).

### 2.2. Measures

#### 2.2.1. STS, Burnout, and Compassion Satisfaction

We used the Japanese Version of the Professional Quality of Life Scale (ProQOL) to assess STS, burnout, and compassion satisfaction [7,28]. The ProQOL consists of 30 items on the STS, burnout, and compassion satisfaction subscales. Respondents rated the frequency of each item related to their experiences in work situations in the past 30 days on a 5-point response scale ranging from 1 (never) to 5 (very often). Sample items include “I get satisfaction from being able to help people” for the compassion satisfaction subscale, “I am happy” for the burnout subscale, and “I jump or am startled by unexpected sounds” for the STS subscale. We calculated the total scores for each subscale. Scores for each subscale range between 10 and 50. Scores between 10 and 21 are considered a low level; scores between 23 and 41 are considered a moderate level, and scores between 42 and 50 are considered a high level [7]. McDonald’s omega coefficients for this study for the burnout, exhaustion, and compassion satisfaction subscales were 0.78, 0.70, and 0.89, respectively.

#### 2.2.2. Coping

We assessed coping strategies using a Brief Version of the Coping Orientation to Problems Experienced Inventory (Brief-COPE) [29,30]. The Brief-COPE is a 28-item self-rating scale comprising 14 subscales related to coping strategies. Respondents rated the perceived frequency of how often they performed each item on a 4-point scale ranging from 1 (I have not been doing this at all) to 4 (I have been doing this a lot). Sample items include “I’ve given up trying to deal with it” and “I’ve been making jokes about it”. Omega coefficients for this study were 0.62 for self-distraction, 0.65 for active coping, 0.78 for denial, 0.89 for substance use, 0.80 for emotional support, 0.79 for behavioral disengagement, 0.68 for venting, 0.86 for instrumental support, 0.64 for reframing, 0.86 for self-blame, 0.74 for planning, 0.78 for humor, 0.70 for acceptance, and 0.50 for religious coping. We conducted an exploratory factor analysis to extract latent factors in these subscales. Factor scores were calculated for each latent factor. Possible values for the factor scores range between −1 and +1. A greater positive value means a higher value for a particular coping factor. We used the factor scores instead of the sum scores because psychometric properties (higher correlations with true scores, higher sensitivity, higher reliability) are generally better for the factor scores than the sum scores in most situations [31].

#### 2.2.3. Empathy

We used the Interpersonal Reactivity Index, Japanese Version (IRI-J), to assess empathy [32]. The IRI-J comprises 28 items that assess empathic concern, personal distress, perspective-taking, and fantasy tendency. Respondents rated the degree to which each statement described their thoughts and feelings in various situations on a 5-point scale ranging from 1 (does not describe me well) to 5 (describes me very well). Sample items include “I often have tender, concerned feelings for people less fortunate than me” for the empathic concern subscale, “In emergency situations, I feel apprehensive and ill-at-ease” for the personal distress subscale, “I try to look at everybody’s side of a disagreement before I make a decision” for the perspective-taking subscale, and “I really get involved with the feelings of the characters in a novel” for the fantasy tendency subscale. We calculated the total score for each subscale. The total scores range between 7 and 35. The higher score means higher levels of empathy for each subscale. Omega coefficients for this study were 0.74 for empathic concern, 0.71 for perspective-taking, 0.81 for personal distress, and 0.81 for the fantasy tendency.

#### 2.2.4. Procedures

All procedures were conducted in accordance with the ethical standards of the relevant national and institutional committees on human research and conformed to the Declaration of Helsinki, as revised in 2013. This study was approved by the Ethics Committee of the National Defense Medical College, Tokorozawa, Japan (Approval No. 4040). We distributed a package containing the questionnaires (paper-and-pencil) to 935 potential participants affiliated with the Japanese Ministry of Defense Hospitals (return rate: 54.1%) between March and May 2020 and March and May 2021. Written informed consent was obtained from all the participants.

#### 2.2.5. Data Analysis

We calculated the Pearson’s correlations among the study variables using the R package, psych (version 2.4.3) [33]. Additionally, we conducted an exploratory factor analysis (EFA) of the 14 coping subscales to extract the underlying structure of coping strategies using the R packages, psych, and GPA rotation (version 2024.2-1) [33,34]. Previous studies disagree with the factor structure of the Brief-COPE [35]. Studies have found that the number of factors ranged from 2 to 14; some studies retained all items, but others excluded items from the factor structure [36]. These findings indicate that the factor structure of the Brief-COPE might not be robust in all situations. Thus, we examined the factor structure of the Brief-COPE in our study. We determined the number of factors using Velicer’s minimum average partial test with the R package and EFA.dimensions (version 0.1.8.1) [36]. We used maximum likelihood estimation as a factor method with the oblique rotation for the EFA.

Finally, we conducted a series of three multiple regression analyses on STS, burnout, and compassion satisfaction as dependent variables using the R packages, car (version 3.1-2) and MASS (version 7.3-60.2) [37,38]. The regression analyses included demographic variables (occupation, age, sex, and marital status), dispositional empathy, and coping (factors identified in the EFA) as independent variables. We estimated 95% bootstrap confidence intervals (CIs; see Supplementary File S1 for the R code used in this study). We followed a reporting approach using confidence intervals and effect sizes but did not depend on *p*-values [39].

#### 2.2.6. Data Handling

Data were missing on age and items on the Brief-COPE and ProQOL. In total, 0.12% of the variables of interest had missing data. A test of missing completely at random (MCAR) showed that missing data were MCAR for the Brief-COPE, χ^2^(27) = 17.41, *p* = 0.921, and the ProQOL, χ^2^(109) = 70.72, *p* = 0.998. These results suggest that missing data occurred randomly without apparent patterns. We imputed missing data using a random forest imputation algorithm with the R package, missForest (version 1.5) [40].

## 3. Results

### 3.1. Exploratory Factor Analysis on Coping

The results of the minimum average partial test indicated three factors in the Brief-COPE subscales. The results of the EFA showed that Factor 1 was related to active coping strategies (variance = 0.18; eigenvalue = 2.50), and Factor 2 comprised subscales related to support seeking (variance = 0.14; eigenvalue = 1.89). Factor 3 consisted mainly of avoidant coping, such as “Denial” and “Substance use,” as well as other coping strategies, such as “Religious coping” and “Humor”, which we termed “indirect coping” (variance = 0.12, eigenvalue = 1.65). Table 2 presents the factor loadings of the Brief-COPE subscales. Subsequent analyses used factor scores for these three factors.

### 3.2. Correlations between the Study Variables and Descriptive Statistics

Table 3 shows the Pearson’s correlations among the study variables. The Pearson’s correlation >0.1 is considered a small effect size; >0.3 is a medium effect size, and >0.5 is a large effect size [41]. Burnout and STS were weakly to moderately related to empathy and coping variables. Moreover, compassion satisfaction was weakly to moderately associated with empathy and coping behavior. The empathy variables were weakly to moderately associated with one another. Table 4 displays the mean and standard deviations for the study variables. Using cutoff criteria for burnout, STS, and compassion satisfaction, 86.5% of the doctors and nurses in our sample had moderate levels of burnout, and 2.3% of them had severe levels of burnout [7]. Additionally, 28.0% experienced moderate levels of STS, and none experienced severe levels of STS. Most doctors and nurses in our sample (81.9%) experienced moderate levels of compassion satisfaction, and 3.9% experienced high levels of compassion satisfaction.

### 3.3. Predicting Burnout, STS, and Compassion Satisfaction (Hypotheses 1 and 2)

#### 3.3.1. Model of Burnout

We performed a series of multiple regression analyses to predict burnout, STS, and compassion satisfaction. Results showed that medical students and nursing students had lower burnout scores than nurses by 3.73 and 2.22, respectively, ranging from −5.27 to −2.20 (95% bootstrap CIs for the unstandardized coefficient) and from –3.55 to −0.89, respectively. When empathic concern scores increased by 1, burnout scores decreased by 0.29, ranging from −0.42 to −0.16 (95% bootstrap CIs for the unstandardized coefficient). In contrast, when personal distress scores increased by 1, burnout scores increased by 0.43, ranging from 0.32 to 0.53 (95% bootstrap CIs for the unstandardized coefficient). Additionally, as active coping scores increased by 1, burnout scores decreased by 1.33, ranging from −0.1.90 to −0.73 (95% bootstrap CIs for the unstandardized coefficient). Conversely, as indirect coping scores increased by 1, burnout scores increased by 0.79, ranging from 0.20 to 1.32 (95% bootstrap CIs for the unstandardized coefficient). The adjusted R-squared was 0.32 for this model, *F*(13, 492) = 19.41, *p* < 0.001. Table 5 displays the unstandardized coefficients, standard errors for the standardized coefficients, standardized coefficients, and 95% bootstrap confidence intervals for the standardized coefficients in the model.

#### 3.3.2. Model on STS

Results showed that medical students had lower STS compared to nurses by 2.28, ranging from −3.66 to −0.99 (95% bootstrap CIs for the unstandardized coefficient). Additionally, as personal distress scores increased by 1, the STS scores increased by 0.28, ranging from 0.19 to 0.37 (95% bootstrap CIs for the unstandardized coefficient). As indirect coping scores increased by 1, the STS scores increased by 1.62, ranging from 1.09 to 2.17 (95% bootstrap CIs for the unstandardized coefficient). The adjusted R-squared was 0.22 for this model; *F*(13, 492) = 12.18; *p* < 0.001.

#### 3.3.3. Model of Compassion Satisfaction

Results of the model of compassion satisfaction showed that medical doctors and medical students had higher scores of compassion satisfaction compared to nurses by 3.27 (ranging from 0.65 to 5.66 [95% bootstrap CIs for the unstandardized coefficient]) and 2.75 (ranging from 0.86 to 4.58), respectively. When empathic concern increased by 1, the compassion satisfaction scores increased by 0.41, ranging from 0.25 to 0.54 (95% bootstrap CIs for the unstandardized coefficient). As fantasy tendency scores increased by 1, compassion satisfaction scores increased by 0.13, ranging from 0.02 to 0.24 (95% bootstrap CIs for the unstandardized coefficient). In contrast, as personal distress increased by 1, compassion satisfaction scores decreased by 0.31, ranging from 0.25 to 0.55 (95% bootstrap CIs for the unstandardized coefficient). Furthermore, when the active coping scores increased by 1, the compassion satisfaction scores increased by 1.77, ranging from 1.05 to 2.47 (95% bootstrap CIs for the unstandardized coefficient). When support-seeking scores increased by 1, compassion satisfaction increased by 0.78, ranging from 0.14 to 1.42 (95% bootstrap CIs for the unstandardized coefficient). The R-squared was 0.30 for this model, *F*(13, 492) = 17.87, *p* < 0.001.

### 3.4. Comparing Empathy between Occupations (Hypothesis 3)

**Figure 1:**
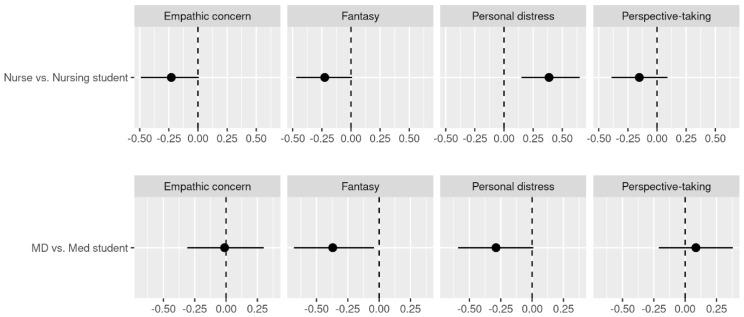
Cohen’s d for the Comparisons Between the Occupations and 95% Bootstrap Confidence Intervals for Cohen’s d. Note. The vertical dotted line indicates Cohen’s d value of zero. The black horizontal lines indicate 95% Bootstrap CIs. The dots indicate the point estimate of Cohen’s d. MD = medical doctor; Med student = medical student; Fantasy = fantasy tendency.

Figure 1 displays Cohen’s d and its 95% bootstrap CIs for comparisons of empathy between nurses and nursing students and between medical doctors and medical students. Nurses reported higher levels of personal distress than nursing students, with a medium effect size (d = 0.39). Medical doctors had a lower fantasy tendency compared to medical students with a medium effect size (d = −0.37).

## 4. Discussion

This study provides data on psychological and behavioral health in relation to STS, burnout, and compassion satisfaction. The data are based on healthcare professionals and students working at Japanese Ministry of Defense Hospitals after the beginning of the COVID-19 pandemic. Compared to the existing data for adult or pediatric healthcare professionals in Japan, fewer doctors and nurses have moderate or high levels of burnout and STS in our sample [4].

The results of the present study reveal that nurses have higher personal distress (an empathy sub-component) than nursing students, and medical doctors have lower fantasy tendency than medical students. Other differences in dispositional empathy show small to medium effect sizes, but their 95% bootstrap confidence intervals crossed zero, indicating that an effect of zero is possible. These results suggest that nurses become more sensitive to others’ distress through patient care and consider it their own distress. Medical doctors might learn about the reality of the medical field after they start practicing, resulting in growing out of an ideal image that they identify with when they are medical students. The results of this study are not entirely consistent with our hypothesis and previous reports that medical and nursing students show a decline in empathy as they graduate and enter the medical profession [17,18,19,20]. These inconsistencies may be due to the interpretation of the results based on the 95% bootstrap confidence intervals of the effect size rather than a point estimate. For example, when examining the point estimate of Cohen’s d, we found slightly lower (small effect sizes) empathic concern and fantasy tendency for nurses compared to nursing students and slightly lower personal distress for physicians compared to medical students.

Our findings indicate that healthcare professionals’ coping behaviors are crucial factors related to burnout, STS, and compassion satisfaction. Active coping is related to lower burnout and higher compassion satisfaction levels; indirect coping is related to higher STS, and support-seeking is related to higher compassion satisfaction. This is consistent with our hypotheses and is similar to a previous finding that active coping and support-seeking are related to the psychological well-being of healthcare professionals [42]. However, the same previous study also found that humor and religious coping were related to psychological well-being. Conversely, our findings indicated that indirect coping, including humor and religious coping, was associated with higher burnout and STS scores. The difference in the results between our study and the previous literature might be attributed to the cultural context of humor and religion in Japan. In general, people in Western countries are more likely to use humor as a coping strategy compared to those in Eastern countries [43]. The meaning of religion is different from Western cultures. The majority of Japanese people think that spirituality is important, but only 30% of them have religious faith [44]. It is difficult to separate the effects of individual coping variables because the indirect coping strategies explored in our study included various coping strategies, such as humor and religious coping, in addition to several avoidant coping strategies. Additionally, a previous study reported findings consistent with those of our study, demonstrating that some of these indirect coping strategies were associated with STS among pediatric nurses [45].

Although our findings are consistent with those of previous studies, the effects of coping behaviors on health are complex. For instance, a previous study found that a variety of coping behaviors, including active coping, denial, emotional support, instrumental support, and self-distraction, were related to higher distress among healthcare professionals working during the 2003 SARS outbreak in Hong Kong [46]. In extremely uncertain situations, people might engage in several different coping behaviors during distress without knowing which is effective.

The findings of the relationship between dispositional empathy and burnout, STS, and compassion satisfaction in our study are consistent with those of previous studies. Our findings showed that empathic concern was related to lower burnout and higher compassion satisfaction. These results are consistent with those of previous studies on nurses and physicians [13,15]. Our findings show that personal distress is related to burnout, STS, and compassion satisfaction, which is consistent with previous studies on cancer healthcare professionals in Ireland, radiation oncologists, Argentinian physicians, and Portuguese nurses [12,13,14,15]. 

Inconsistent with our findings, a study indicated that higher empathic concern is related to higher burnout among Argentinian physicians [13]. Additionally, higher empathic concern is related to higher STS among Argentinian physicians and cancer healthcare professionals [13,14]. These inconsistent findings regarding the relationship between empathy, burnout, and STS could be due to several factors, such as occupation, culture, career length, and the unique situations experienced by healthcare professionals. Our findings and previous studies show that personal distress, one of the constructs of empathy traits, is consistently related to elevated burnout and STS. Thus, a psychoeducational intervention that cultivates empathy and promotes awareness of the relationship between personal distress, burnout, and STS decreases personal distress, reinforces active coping strategies, and may be helpful in enhancing the psychological health of healthcare professionals.

In the job demands and resources model of burnout, empathic concern, active coping, and support-seeking coping strategies are potential factors that could prevent burnout and STS and facilitate compassion satisfaction among healthcare professionals [47]. Based on the job demands and resources model, the effects of these factors need to be stronger than those of job demands, such as workload, time pressure, and inadequate work environments. Job demands can be more difficult to alter because of the occasional requirements of organizational change, but empathy and coping strategies might be more amenable at the individual level. 

### 4.1. Practical Implication

Our findings indicate that sub-components of empathy differently influence burnout, STS, and compassion satisfaction. These results suggest that simply cultivating all aspects of human empathy lumped together might not improve the well-being of healthcare professionals. For example, healthcare professionals might be able to prevent burnout and STS more if they could lower their personal distress. Moreover, they might benefit from increasing their empathic concern to prevent burnout and enhance compassion satisfaction. Previous studies reported psychological interventions aiming at fostering empathy, but these interventions did not necessarily focus on decreasing their personal distress (e.g., [48,49]). It is suggested that more precise psycho-educational interventions that incorporate these perspectives may be more effective in reducing burnout and STS among healthcare professionals. Additionally, the current study suggests that a student population can be a suitable target for an intervention. Although some previous studies reported the results of a randomized controlled trial on nursing or medical students (e.g., [50,51]), they usually measured post-intervention empathy when participants were still students. Thus, further study is needed to confirm whether such interventions for students would be effective in preventing burnout or STS and increasing compassion satisfaction even after they become healthcare professionals.

### 4.2. Limitations

This study has certain limitations. Our study is cross-sectional; hence, it is not possible to determine causal relationships. In the future, we will follow up with participants to longitudinally investigate the relationship between coping strategies and psychological responses among Japanese healthcare professionals. Although this study provided critical data on the relationship between empathy and burnout, STS, and compassion satisfaction, our findings do not resolve inconsistent findings on this topic. Future studies should attempt to resolve these discrepancies.

Despite these limitations, this study provides important data on the relationships between empathy, coping, burnout, STS, and compassion satisfaction among Japanese healthcare professionals. Our findings may be useful for psychoeducational interventions aimed at improving the psychological well-being of healthcare professionals.

### 4.3. Conclusions

The current study is part of a longitudinal study examining the trajectories of empathy and their effects on psychological distress among young Japanese healthcare workers and medical and nursing students. This study conducted cross-sectional analyses using the baseline data. Our findings indicate that sub-components of empathy function differently with burnout, STS, or compassion satisfaction. Particularly, personal distress is related to higher burnout and STS and lower compassion satisfaction. Furthermore, our results suggest the importance of coping strategies. Active coping is associated with lower burnout and higher compassion satisfaction; support-seeking is related to higher compassion satisfaction, and indirect coping is related to higher burnout and STS. Components of empathy among healthcare professionals were speculated to change in complex ways as their careers progressed. We found that nurses had higher personal distress than nursing students, and medical doctors had lower fantasy tendencies than medical students. Even though the current study has limitations, such as the cross-sectional design, we showed the baseline of young healthcare professionals and medical and nursing students who will soon be healthcare professionals. These findings could be useful information in developing more precise psycho-educational interventions to prevent burnout and STS among healthcare professionals.

## Figures and Tables

**Table 1:** Demographics of the Participants.

		Doctor (*n* = 181)		Nurse (*n* = 123)		Medical Student (*n* = 63)		Nursing Student (*n* = 139)	
Variable	Levels	Count	%	Count	%	Count	%	Count	%
Gender									
	Female	51	28.2	115	93.5	30	47.6	129	92.8
	Male	130	71.8	8	6.5	33	52.4	10	7.2
Marital status									
	Married	87	48.1	11	8.9	3	4.8	0	0.0
	Single	94	51.9	112	91.1	60	95.2	139	100.0

**Table 2:** Factor Loadings of the Brief-COPE Items.

Items	Active Coping	Support Seeking	Indirect Coping
Planning	0.82	0.04	0.01
Active coping	0.74	0.08	−0.03
Positive reframing	0.64	−0.05	0.21
Acceptance	0.55	0.08	−0.17
Emotional support	−0.05	0.94	0.00
Instrumental support	0.22	0.74	−0.03
Venting	−0.08	0.55	0.16
Denial	−0.13	0.07	0.65
Religious coping	0.26	−0.01	0.51
Humor	0.22	−0.08	0.47
Substance use	−0.11	0.11	0.43
Behavioral disengagement	−0.52	0.01	0.42
Self-distraction	−0.04	0.24	0.37
Self-blaming	0.17	−0.05	0.33

Note. Values in bold indicate items of the same factor.

**Table 3:** Pearson’s Correlation Coefficients and Their 95% Confidence Intervals Between the Study Variables.

	**1**	**2**	**3**	**4**	**5**
1. Empathic concern					
2. Personal distress	0.19				
	[0.10, 0.27]				
3. Perspective taking	0.35	−0.15			
	[0.27, 0.42]	[−0.23, −0.06]			
4. Fantasy tendency	0.33	0.29	0.17		
	[0.25, 0.41]	[0.21, 0.37]	[0.09, 0.25]		
5. Burnout	−0.19	0.39	−0.13	−0.02	
	[−0.27, −0.10]	[0.31, 0.46]	[−0.22, −0.05]	[−0.10, 0.07]	
6. STS	0.12	0.35	0.06	0.15	0.38
	[0.03, 0.20]	[0.27, 0.43]	[−0.03, 0.15]	[0.06, 0.23]	[0.30, 0.45]
7. CS	0.30	−0.24	0.22	0.12	−0.68
	[0.22, 0.37]	[−0.32, −0.16]	[0.13, 0.30]	[0.03, 0.20]	[−0.73, −0.63]
8. Active coping	0.25	−0.22	0.35	0.08	−0.39
	[0.17, 0.33]	[−0.30, −0.14]	[0.27, 0.42]	[−0.01, 0.17]	[−0.47, −0.32]
9. Support seeking	0.27	0.14	0.00	0.16	−0.09
	[0.19, 0.35]	[0.06, 0.23]	[−0.9, 0.09]	[0.07, 0.24]	[−0.17, 0.00]
10. Indirect coping	0.00	0.27	0.01	0.19	0.26
	[−0.09, 0.09]	[0.19, 0.35]	[−0.8, 0.09]	[0.11, 0.28]	[0.18, 0.34]
	**6**	**7**	**8**	**9**	
7. CS	−0.14				
	[−0.22, −0.05]				
8. Active coping	−0.12	0.43			
	[−0.20, −0.03]	[0.36, 0.50]			
9. Support seeking	0.10	0.18	0.34		
	[0.01, 0.19]	[0.10, 0.27]	[0.26, 0.42]		
10. Indirect coping	0.36	−0.18	−0.30	0.17	
	[0.28, 0.43]	[−0.26, −0.09]	[−0.37, −0.22]	[0.09, 0.26]	

Note. STS, secondary traumatic stress. CS, compassion satisfaction. The values in square brackets indicate the 95% confidence intervals.

**Table 4:** Means and Standard Deviations for the Study Variables.

	Doctor (*n* = 181)		Nurse (*n* = 123)		Medical Student (*n* = 63)		Nursing Student (*n* = 139)	
Variable	Mean	SD	Mean	SD	Mean	SD	Mean	SD
Empathic concern	22.96	4.00	23.77	3.71	23.02	5.00	24.71	4.41
Personal distress	20.75	4.93	23.51	4.59	22.30	5.80	21.72	4.69
Perspective taking	22.83	4.08	22.07	3.97	22.44	4.86	22.70	4.25
Fantasy tendency	21.25	4.77	22.24	5.56	23.25	6.00	23.50	5.56
Burnout	27.76	5.85	30.54	5.76	26.25	5.18	27.14	5.65
STS	18.94	4.89	19.85	5.23	17.14	4.44	18.63	4.46
CS	30.65	7.05	26.41	6.50	29.27	6.31	29.08	6.79
Active coping	0.05	1.02	−0.21	0.84	0.04	0.88	0.10	0.98
Support seeking	−0.20	0.94	0.25	0.81	−0.37	0.98	0.21	1.01
Indirect coping	−0.12	0.87	0.08	0.85	0.12	0.95	0.03	0.90

Note. Active coping, support-seeking, and indirect coping were the factor scores. SD, standard deviation; STS, secondary traumatic stress; CS, compassion fatigue.

**Table 5:** Unstandardized Coefficients, Standard Errors for Standardized Coefficients, Standardized Coefficients, and 95% Bootstrap Confidence Intervals for Standardized Coefficients in the Regression Analyses.

DV	IV	B	SE (β)	β	95% CI LL	95% CI UL
Burnout						
	Intercept	29.11	0.15	−0.39	−0.68	−0.11
	MD (vs. Nurse)	−0.75	0.17	−0.51	0.19	0.83
	Nursing student (vs. Nurse)	−2.22	0.15	−0.26	−0.02	0.53
	Med. student (vs. Nurse)	−3.73	0.14	−0.64	0.37	0.91
	Men (vs. Women)	−0.16	0.10	−0.03	−0.21	0.17
	Marriage—single (vs. Married)	−0.05	0.12	−0.01	−0.25	0.24
	Age	−0.14	0.09	−0.08	−0.26	0.09
	Empathic concern	−0.29	0.04	−0.21	−0.30	−0.12
	Personal distress	0.43	0.04	0.36	0.27	0.45
	Perspective-taking	0.12	0.04	0.09	−0.01	0.18
	Fantasy tendency	−0.06	0.04	−0.06	−0.15	0.04
	Active coping	−1.33	0.05	−0.22	−0.32	−0.12
	Support seeking	−0.33	0.05	−0.05	−0.15	0.04
	Indirect coping	0.79	0.04	0.12	0.04	0.20
STS						
	Intercept	6.64	0.16	−0.31	−0.61	0.02
	MD (vs. Nurse)	−0.25	0.19	−0.42	0.10	0.75
	Nursing student (vs. Nurse)	−0.45	0.16	−0.38	0.08	0.67
	Med. student (vs. Nurse)	−2.28	0.15	−0.47	0.19	0.75
	Men (vs. Women)	−0.75	0.11	−0.15	−0.37	0.07
	Marriage—single (vs. Married)	−0.02	0.13	−0.004	−0.25	0.24
	Age	0.14	0.10	0.10	−0.09	0.28
	Empathic concern	0.04	0.05	0.04	−0.06	0.13
	Personal distress	0.28	0.05	0.29	0.20	0.40
	Perspective-taking	0.10	0.05	0.09	0.003	0.18
	Fantasy tendency	0.002	0.04	0.003	−0.08	0.11
	Active coping	0.07	0.05	0.01	−0.08	0.11
	Support seeking	−0.16	0.05	−0.03	−0.12	0.06
	Indirect coping	1.62	0.05	0.30	0.20	0.40
CS						
	Intercept	19.87	0.15	0.12	−0.17	0.43
	MD (vs. Nurse)	3.27	0.17	0.07	−0.29	0.43
	Nursing student (vs. Nurse)	1.23	0.15	0.22	−0.51	0.07
	Med. student (vs. Nurse)	2.75	0.14	0.40	−0.65	−0.12
	Men (vs. Women)	−0.13	0.10	−0.02	−0.22	0.20
	Marriage—single (vs. Married)	0.13	0.11	0.02	−0.23	0.25
	Age	0.09	0.09	0.05	−0.13	0.23
	Empathic concern	0.41	0.04	0.25	0.16	0.34
	Personal distress	−0.31	0.04	−0.23	−0.31	−0.14
	Perspective-taking	−0.03	0.04	−0.02	−0.11	0.07
	Fantasy tendency	0.13	0.04	0.10	0.01	0.19
	Active coping	1.77	0.05	0.24	0.15	0.35
	Support seeking	0.78	0.05	0.11	0.02	0.20
	Indirect coping	−0.52	0.04	−0.07	−0.15	0.01

Note. Job type, gender, marital status are categorical variables. Doctors, nursing students, and nurses were compared with medical students. Men were compared with women. Single was compared with married. DV = dependent variable; IV = independent variable; B = unstandardized coefficient; SE = standard error; β = standardized coefficients; CI = confidence interval; LL = lower limit; UL = upper limit; MD = medical doctor; STS = secondary traumatic stress; CS = compassion satisfaction.

## Data Availability

The data for this study are available upon request.

## Supplementary Materials

The following supporting information can be downloaded at: https://www.mdpi.com/article/10.3390/bs14050400/s1, File S1: R commands; File S2: Questionnaires.
