# Supplementary material for: Empathy and Coping Strategies Predict Quality of Life in Japanese Healthcare Professionals
Source: Behav Sci (Basel). 2024 May 11;14(5):400. doi: 10.3390/bs14050400 (PMC11117590; doi:10.3390/bs14050400)
Supplement: Supplementary file 1 [file behavsci-14-00400-s001.zip › behavsci-2980162-supplementary.pdf]

Supplementary File S1 (R commands)

```
#========================efa=================================================
##make correlation matrix.
library(EFA.dimensions)

cor = POLYCHORIC_R(imp2[,109:122], )

##velicer's map test.
library(EFA.dimensions)

MAP(cor)

##efa
library(psych)
library(GPArotation)

efa = fa(imp2[,109:122], nfactors=3, fm='ml', rotate='oblimin', cor='poly',
n.obs=506)
print(efa, sort=T)

imp2$active_cope = efa$scores[,1]
imp2$suppseek_cope = efa$scores[,2]
imp2$passive_cope = efa$scores[,3]
names(imp2)

#================================correlations=================================
library(psych)

cor = corr.test(imp2[,c(102:108,123:125)], method='pearson', adjust='none',
                alpha=.05, ci=T)

#==========================difference in occupations==========================
library(rstatix)
library(ggplot2)
library(stringr)

ec_d = cohens_d(data=imp2, iri_ec~role, ci=T, conf.level=.95, ci.type='bca',
                nboot=5000)
TukeyHSD(aov(iri_ec~role, data=imp2))
ec_d$Comparison = str_c(ec_d$group1, '*', ec_d$group2)
ec_d$ES = 'ES_EC'

pd_d = cohens_d(data=imp2, iri_pd~role, ci=T, conf.level=.95, ci.type='bca',
                nboot=5000)
TukeyHSD(aov(iri_pd~role, data=imp2))
pd_d$Comparison = str_c(pd_d$group1, '*', pd_d$group2)
pd_d$ES = 'ES_PD'

pt_d = cohens_d(data=imp2, iri_pt~role, ci=T, conf.level=.95, ci.type='bca',
                nboot=5000)
TukeyHSD(aov(iri_pt~role, data=imp2))
pt_d$Comparison = str_c(pt_d$group1, '*', pt_d$group2)
pt_d$ES = 'ES_PT'

fs_d = cohens_d(data=imp2, iri_fs~role, ci=T, conf.level=.95, ci.type='bca',
                nboot=5000)
TukeyHSD(aov(iri_fs~role, data=imp2))
```

```
fs_d$Comparison = str_c(fs_d$group1, '*', fs_d$group2)
fs_d$ES = 'ES_FS'

ES = rbind(ec_d, pd_d, pt_d, fs_d)

library(dplyr)

ES <- ES %>%
  rename(Cohens_d=effsize)

es_labs = c("Empathic concern","Fantasy","Personal
distress","Perspective-taking")
names(es_labs) = c("ES_EC", "ES_FS","ES_PD","ES_PT")

ES_select = subset(ES, group1=='nurse' & group2=='nurse_student')

plt1 = ggplot(ES_select[c(1:4),], aes(Cohens_d, y=Comparison)) +
       geom_pointrange(aes(xmin=conf.low, xmax=conf.high)) +
       facet_wrap(~ES, ncol=4, labeller=labeller(ES=es_labs)) +
       labs(x="", y="") +
       scale_y_discrete(labels=c("nurse*nurse_student"="Nurse vs. Nursing
student")) +
       geom_vline(aes(xintercept = 0),linetype="dashed")

ES_select = subset(ES, group1=='MD' & group2=='med_student')

plt2 = ggplot(ES_select[c(1:4),], aes(Cohens_d, y=Comparison)) +
  geom_pointrange(aes(xmin=conf.low, xmax=conf.high)) +
  facet_wrap(~ES, ncol=4, labeller=labeller(ES=es_labs)) +
  labs(x="", y="") +
  scale_y_discrete(labels=c("MD*med_student"="MD vs. Med student")) +
  geom_vline(aes(xintercept = 0),linetype="dashed")

library(patchwork)

lo = '
      AAAA
      BBBB
     '

plt1 + plt2 + plot_layout(design=lo)

ggsave('Figure1.tiff', dpi=300, units='cm', width=22, height=10)

#==============================regression=====================================
library(car)
library(MASS)

#burnout
burnout_lm = lm(burnout~role+gender+marriage+age+iri_ec+iri_pd+iri_pt
                   +iri_fs+active_cope+suppseek_cope+passive_cope, data=imp2)
summary(burnout_lm)

set.seed=101
boot_bo = Boot(burnout_lm, f=coef, R=5000, method='case')
conf_bo = confint(boot_bo, level = .95)

#STS
exhaustion_lm = lm(exhaustion~role+gender+marriage+age+iri_ec+iri_pd+iri_pt
```

```
                       +iri_fs+active_cope+suppseek_cope+passive_cope, data=imp2)
summary(exhaustion_lm)

boot_sts = Boot(exhaustion_lm, f=coef, R=5000, method='case')
conf_sts = confint(boot_sts, level=.95)

#compassion satisfaction.
jsatisfaction_lm = lm(jsatisfaction~role+gender+marriage+age+iri_ec
                       +iri_pd+iri_pt+iri_fs+active_cope
                       +suppseek_cope+passive_cope, data=imp2)
summary(jsatisfaction_lm)

boot_js = Boot(jsatisfaction_lm, f=coef, R=5000, method='case')
conf_js = confint(boot_js, level=.95)

#==================================mean, sd====================================
library(psych)

summ = describeBy(imp2[,c(106:108,102:105,123:125)], imp2$role, mat=T)
write.csv(summ, file='summary.csv')

#==============================cutoff==========================================
hp = subset(imp2, role=='MD' | role=='Nurse')

nrow(subset(hp, burnout>=42))/304
nrow(subset(hp, burnout>=22 & burnout<=41))/304
nrow(subset(hp, burnout<=21))/304
nrow(subset(hp, exhaustion>=42))/304
nrow(subset(hp, exhaustion>=22 & exhaustion<=41))/304
nrow(subset(hp, exhaustion<=21))/304
nrow(subset(hp, jsatisfaction>=42))/304
nrow(subset(hp, jsatisfaction>=22 & exhaustion<=41))/304
nrow(subset(hp, jsatisfaction<=21))/304
```

Supplementary File S2 (Questionnaires)

Professional Quality of Life Scale (ProQOL)

When you [help] people you have direct contact with their lives. As you may have found, your compassion for those you [help] can affect you in positive and negative ways. Below are some questions about your experiences, both positive and negative, as a [helper]. Consider each of the following questions about you and your current work situation. Select the number that honestly reflects how frequently you experienced these things in the last 30 days.

1 = Never, 2 = Rarely, 3 = Sometimes, 4 = Often, 5 = Very Often

1. I am happy.

2. I am preoccupied with more than one person I [help].

3. I get satisfaction from being able to [help] people.

4. I feel connected to others.

5. I jump or am startled by unexpected sounds.

6. I feel invigorated after working with those I [help].

7. I find it difficult to separate my personal life from my life as a [helper].

8. I am not as productive at work because I am losing sleep over traumatic experiences of a person I [help].

9. I think that I might have been affected by the traumatic stress of those I [help].

10. I feel trapped by my job as a [helper].

11. Because of my [helping], I have felt "on edge" about various things.

12. I like my work as a [helper].

13. I feel depressed because of the traumatic experiences of the people I [help].

14. I feel as though I am experiencing the trauma of someone I have [helped].

15. I have beliefs that sustain me.

16. I am pleased with how I am able to keep up with [helping] techniques and protocols.

17. I am the person I always wanted to be.

18. My work makes me feel satisfied.

19. I feel worn out because of my work as a [helper].

20. I have happy thoughts and feelings about those I [help] and how I could help them.

21. I feel overwhelmed because my case [work] load seems endless.

22. I believe I can make a difference through my work.

23. I avoid certain activities or situations because they remind me of frightening experiences of the people I [help].

24. I am proud of what I can do to [help].

25. As a result of my [helping], I have intrusive, frightening thoughts.

26. I feel "bogged down" by the system.

27. I have thoughts that I am a "success" as a [helper].

28. I can't recall important parts of my work with trauma victims.

29. I am a very caring person.

30. I am happy that I chose to do this work.

Brief - Coping Orientation to Problems Experienced Inventory (Brief-COPE)

Instructions:

The following questions ask how you have sought to cope with a hardship in your life. Read the statements and indicate how much you have been using each coping style.

1 = I haven't been doing this at all, 2 = A little bit, 3 = A medium amount, 4 = I've been doing this a lot

1. I've been turning to work or other activities to take my mind off things.
2. I've been concentrating my efforts on doing something about the situation I'm in.
3. I've been saying to myself "this isn't real".
4. I've been using alcohol or other drugs to make myself feel better.
5. I've been getting emotional support from others.
6. I've been giving up trying to deal with it.
7. I've been taking action to try to make the situation better.
8. I've been refusing to believe that it has happened.
9. I've been saying things to let my unpleasant feelings escape.
10. I've been getting help and advice from other people.
11. I've been using alcohol or other drugs to help me get through it.
12. I've been trying to see it in a different light, to make it seem more positive.
13. I've been criticizing myself.
14. I've been trying to come up with a strategy about what to do.
15. I've been getting comfort and understanding from someone.
16. I've been giving up the attempt to cope.
17. I've been looking for something good in what is happening.
18. I've been making jokes about it.
19. I've been doing something to think about it less, such as going to movies, watching TV, reading, daydreaming, sleeping, or shopping.
20. I've been accepting the reality of the fact that it has happened.
21. I've been expressing my negative feelings.
22. I've been trying to find comfort in my religion or spiritual beliefs.
23. I've been trying to get advice or help from other people about what to do.
24. I've been learning to live with it.

25. I've been thinking hard about what steps to take.
26. I’ve been blaming myself for things that happened.
27. I've been praying or meditating.
28. I've been making fun of the situation.

Interpersonal Reactivity Index

The following statements inquire about your thoughts and feelings in a variety of situations. For each item, indicate how well it describes you by choosing the appropriate letter on the

scale at the top of the page: 1, 2, 3, 4, or 5. When you have decided on your answer, fill in the letter next to the item number. READ EACH ITEM CAREFULLY BEFORE RESPONDING. Answer as honestly as you can. Thank you.

1	2	3	4	5
Does not describe me well				Describes very well

1. I daydream and fantasize, with some regularity, about things that might happen to me.
2. I often have tender, concerned feelings for people less fortunate than me.
3. I sometimes find it difficult to see things from the "other guy's" point of view.
4. Sometimes I don't feel very sorry for other people when they are having problems.
5. I really get involved with the feelings of the characters in a novel.
6. In emergency situations, I feel apprehensive and ill-at-ease.
7. I am usually objective when I watch a movie or play, and I don't often get completely caught up in it.
8. I try to look at everybody's side of a disagreement before I make a decision.
9. When I see someone being taken advantage of, I feel kind of protective towards them.
10. I sometimes feel helpless when I am in the middle of a very emotional situation.
11. I sometimes try to understand my friends better by imagining how things look from their perspective.
12. Becoming extremely involved in a good book or movie is somewhat rare for me.
13. When I see someone get hurt, I tend to remain calm.
14. Other people's misfortunes do not usually disturb me a great deal.
15. If I'm sure I'm right about something, I don't waste much time listening to other people's arguments.
16. After seeing a play or movie, I have felt as though I were one of the characters.
17. Being in a tense emotional situation scares me.
18. When I see someone being treated unfairly, I sometimes don't feel very much pity for them.
19. I am usually pretty effective in dealing with emergencies.
20. I am often quite touched by things that I see happen.
21. I believe that there are two sides to every question and try to look at them both.
22. I would describe myself as a pretty soft-hearted person.
23. When I watch a good movie, I can very easily put myself in the place of a leading character.
24. I tend to lose control during emergencies.
25. When I'm upset at someone, I usually try to "put myself in his shoes" for a while.

26. When I am reading an interesting story or novel, I imagine how I would feel if the events in the story were happening to me.
27. When I see someone who badly needs help in an emergency, I go to pieces.
28. Before criticizing somebody, I try to imagine how I would feel if I were in their place.
